# Depressive Symptoms among Economically Disadvantaged African American Older Adults in South Los Angeles

**DOI:** 10.3390/brainsci9100246

**Published:** 2019-09-22

**Authors:** Meghan C. Evans, Sharon Cobb, James Smith, Mohsen Bazargan, Shervin Assari

**Affiliations:** 1 Department of Family Medicine, Charles R Drew University of Medicine and Science, Los Angeles, CA 90095, USA; meg.e.carlsen@gmail.com (M.C.E.); jamessmith@cdrewu.edu (J.S.); 2 School of Nursing, Charles R Drew University of Medicine and Science, Los Angeles, CA 90095, USA; sharoncobb1@cdrewu.edu; 3 Department of Family Medicine, Charles R Drew University of Medicine and Science, Los Angeles, CA 90095, USA; mohsenbazargan@cdrewu.edu; 4 Department of Family Medicine, University of California Los Angeles (UCLA), Los Angeles, CA 90095, USA

**Keywords:** race, blacks, African Americans, ethnic groups, depression, depressive symptoms

## Abstract

Background: Although social, behavioral, and health factors correlate with depressive symptoms, less is known about these links among economically disadvantaged African American (AA) older adults. Objective: To study social, behavioral, and health correlates of depressive symptoms among economically disadvantaged AA older adults. Methods: This survey was conducted in South Los Angeles between 2015 and 2018. A total number of 740 AA older adults (age ≥55 years) were entered to this study. Independent variables were gender, age, educational attainment, financial difficulties, living alone, marital status, smoking, drinking, chronic medical conditions (CMCs), and pain intensity. The dependent variable was depressive symptoms. Linear regression model was used to analyze the data. Results: Age, financial difficulties, smoking, CMCs, and pain intensity were associated with depressive symptoms. Gender, educational attainment, living arrangement, marital status, and drinking were not associated with depressive symptoms. Conclusion: Factors such as age, financial difficulties, smoking, CMCs, and pain may inform programs that wish to screen high risk economically disadvantaged AA older adults for depressive symptoms.

## 1. Introduction

While a variety of social, behavioral, and health factors contribute to and correlate with depression [1,2,3,4,5,6,7,8], certain factors and their influence may vary across race/ethnicity [9,10,11]. Research has found a Black-White paradox concerning depression wherein African Americans (AAs) have a disproportionately high exposure to psychological distress but lower prevalence rates of depression across major epidemiological studies [12]. However, the National Health and Nutrition Examination Survey recently showed AAs have a higher rate of depression (9.2%) than Hispanic (8.2%), non-Hispanic White (7.9%), and non-Hispanic Asian (3.1%) populations [13].

Additionally, despite the Black-White paradox in depression rates, research has consistently found that depressive symptoms are more prevalent in AA populations [14,15,16]. Research has also demonstrated higher chronicity and severity of depressive symptoms among AA populations compared to Whites [17]. As with other conditions, AAs face disparities in diagnosis and treatment for depression [18,19]. Research has often found that factors, such as socioeconomic status, gender, and health behaviors, are associated with depression across the general population [4,20,21]. Yet, it is imperative to understand how these factors influence the risk for depressive symptoms among AA adults to direct appropriate intervention and treatment for a population at heightened risk. 

This research will add to the existing literature by examining the relationship between a variety of social, behavioral, and health factors and depressive symptoms specifically among AA older adults in economically disadvantaged area of Los Angeles, with the highest percentage of households living below 100% of the Federal Poverty Level (FPL) in the county [22]. These factors include: gender, age, socioeconomic status indicators (educational attainment and financial difficulties), living arrangements and marital status, health behaviors (smoking and drinking), and chronic medical conditions and pain.

### 1.1. Gender, Age, and Depressive Symptoms 

A majority of the existing research has demonstrated gender differences in depressive symptoms and types of diagnoses [1,2]. The Centers for Disease Control and Prevention (CDC) reports the prevalence of depression among women is almost twice as much as men (10.4% and 5.5% respectively) [13]. Gender differences in depressive symptoms may operate differently in AA populations. While research has found depressive symptoms to be higher among AA women [23], other findings reveal no differences in depressive symptom rates between AA men and women [14]. AA women have shown more depressive symptoms than White women [24], but recent research has found that AA men have similar odds of reporting depressive symptoms as White men [25]. A review of the literature by Walton and Payne [26] shows differences in socialization and the stressors that face AA men and AA women could lead to different presentations of depressive symptoms as well as differences in treatment of those symptoms.

Age also plays a role in the development, diagnosis, and treatment of depression. Older age is an important risk factor for poor outcomes related to major depressive disorder (MDD) [3]. Research on health-related quality of life (HRQOL) found an age and gender interaction in which age was strongly associated with better physical component scores for AA men but not for AA women [27]. However, older age was associated with better mental component scores for both AA men and women [27]. Despite this positive effect for older AA adults, other research has shown that stigma plays a major role keeping depressed older adults from seeking mental health treatment. Specifically, AA older adults reveal more internalized stigma and negative attitudes toward mental health treatment than White older adults [28]. The complicated relationship between gender, age, and depressive symptoms specifically among AA adults is important to parse in order to identify and understand vulnerable populations. 

### 1.2. Socioeconomic Status and Depressive Symptoms

Socioeconomic status (SES) factors, including lower educational attainment and financial difficulties, have been associated with depressive symptoms [4,29,30]. While educational attainment has been identified as a potential protective factor against depression [31], other research indicates that these SES factors may not function the same across racial/ethnic groups. Assari, Preiser, and Kelly [32] found that educational attainment is associated with increases in future income and subsequent increases in emotional well-being for Whites but not AAs. Furthermore, higher educational attainment is more protective against depressive symptoms and psychological distress for AA women compared to AA men [33]. In terms of educational attainment, 19% of AA young adults have less than a high school education compared to 11% of White young adults [34]. Additionally, financial distress is strongly associated with major depressive episodes for AA adults as compared to White adults [9]. Furthermore, 22% of AAs live below the poverty line compared to 8.8% of Whites [35]. Considering the disparities in educational and financial attainment, it is critical to understand the role of SES indicators in the risk for depressive symptoms among AA adults. 

In addition to racial/ethnic and gender differences in the relationship between SES and depressive symptoms, research also indicates that SES may influence depression differently across the lifespan [36]. SES inequalities appear to widen across the life course with differences in depression incidence being wider in late adulthood, indicating an increased risk for older adults with low SES to develop symptoms [36]. It was therefore important to uncover the role of SES in the risk of depressive symptoms in our sample of older AA adults. 

### 1.3. Living Arrangements and Depressive Symptoms

Literature has demonstrated that living alone is associated with depressive symptoms, especially in older adults [5,37,38], often due to a lack of sense of belonging and social isolation [39,40]. Marriage, on the other hand, has typically been associated with lower depression scores [6,41]. For AA parents specifically, higher levels of support from a spouse predicted larger decreases in depressive symptoms [42]. Furthermore, a meta-analysis on this relationship in people over the age of 55 revealed that married older people had a lower risk of depression compared to unmarried and divorced older people [43].

There is a lack of studies related to depressive distress and marital status among AA older adults. Brown, Gary, Greene, and Milburn [44] find that social support and affiliations does not buffer against depressive symptoms for AA adults. However, more recent research shows that social support from church and family protect against depressive symptoms for older AA adults [45]. Considering the theoretical understanding that the social and instrumental support derived from marriage versus living alone may protect against stress and depressive symptoms, it is imperative to understand whether this relationship holds true in an AA population who have greater exposure to stressors.

### 1.4. Health Behaviors and Depressive Symptoms

The prevalence of certain health behaviors, such as tobacco and alcohol use, has often been comorbid with depressive symptoms. A nationally representative study revealed that the current smoking rate for those with past-month mental illness was 41% compared to 22.5% of those with no mental illness [46]. Additionally, a recent meta-analysis revealed that depression and smoking are associated across a variety of moderators [47]. Similarly, much of the research has found a relationship between alcohol use or alcohol use disorder (AUD) and depression, such that increased alcohol use increases the risk for depression [7,48]. 

While according to CDC data, AA adults are about equally as likely to be current smokers as White adults (20.2% and 20.8% prevalence respectively) [49], AAs have the highest incidence rate of lung cancer [50] as well as higher exposure to other stressors, such as financial difficulties, which have been linked with smoking and alcohol consumption [51,52]. CDC data also indicates that White men are more likely to be current regular drinkers than AA men (65.5% and 52.9% respectively) and White women are more likely to be current regular drinkers than AA women (55.6% and 35.9% respectively) [53]. However, AA men have a higher rate of alcohol-related injuries/accidents as well as health and social consequences, and AA women have a higher risk of alcohol dependence compared to their White counterparts [10]. Considering the complicated relationship AA adults have with smoking and alcohol use, it is important to include these health behaviors in our model of potential predictors of depressive symptoms. 

### 1.5. Chronic Medical Conditions/Pain and Depressive Symptoms

According to past research, the relationship between depressive symptoms and chronic medical conditions (CMCs) and pain is complex. While there is often a bidirectional relationship between depressive symptoms and CMCs or pain intensity [54,55,56], this relationship differs across racial/ethnic groups. While AA adults have a higher prevalence of CMCs [8,11], studies have often documented a Black-White paradox where AA populations have greater exposure to distress and CMCs but lower rates of mental health conditions such as depression [12,57].

Still, previous studies have indicated higher chronicity of depression for AA adults compared to Whites [58] as well as higher odds of CMCs for AA adults with depressive symptoms [8,11]. For example, González and Tarraf [59] found from a national probability sample that AAs are most likely to meet criteria for comorbid MDD and cardiovascular disease (CVD), and AAs also have the highest disease burden of depression. Not only can CMCs increase risk for depression, but depression can increase the risk of certain CMCs, such as diabetes, which in turn increases the risk of CVD and premature mortality [56]. Research further finds that AAs have higher odds of comorbid MDD with hypertension, obesity, and liver disease than Whites and finds evidence for racial differences in depressive symptoms, disability, and receipt of services [60]. Pain intensity is also associated with more depressive symptoms for AAs than for Whites [61], and less treatment for pain is given to racial/ethnic minorities than to White patients [62]. Given the heightened probability of CMCs and pain in older adults and in AA populations, it is vital to understand how these conditions and experiences in our sample influence the risk of depression which may go undertreated or heighten the adverse effects of CMCs and pain intensity.

### 1.6. Current Study

To better understand predictors of depressive symptoms in economically disadvantaged AA older adults [63,64,65,66], this study explored the roles of gender, age, SES indicators (educational attainment and financial difficulties), living alone, marital status, smoking, drinking, CMCs, and pain intensity on depressive symptoms. 

## 2. Methods

### 2.1. Design and Setting

This study consisted of a cross-sectional survey of AA older adults in economically disadvantaged areas of South Los Angeles, performed between 2015 and 2018.

### 2.2. Institutional Review Board (IRB)

The Institutional Review Board (IRB) of the Charles R. Drew University of Medicine and Science (CDU), Los Angeles approved this study protocol. Before being enrolled in this study, all participants signed a written informed consent and received a financial incentive following their enrollment. 

### 2.3. Process and Data Collection 

Data was collected through structured face-to-face interviews concerning demographic factors (gender and age), SES (educational attainment and financial difficulty), living alone, marital status, health behaviors (smoking and drinking), health (CMCs and pain intensity), and depressive symptoms.

### 2.4. Participants

The study used convenience sampling to recruit AA older adults from economically disadvantaged areas in the South Los Angeles, such as the Compton and Watts area. The study included participants from Service Planning Area (SPA) 6 as one of the most economically disadvantaged urban areas in Los Angeles County, with the lowest median income ($ 36,400), the highest unemployment rate (13.6%), and the highest percentage of household incomes less than 100% of the FPL (33.6%) [22,67]. Furthermore, SPA6 also has the highest percentage of AAs of SPAs in Los Angeles (27.4%) [22]. Eligibility was limited to older adults who were AA, 55 years or older, could complete an interview in English, and resided in SPA6. Exclusion criteria included institutionalization, enrollment in any other clinical trials, or poor cognitive performance. This sampling resulted in 740 AAs adults aged 55 years and older. 

### 2.5. Measures

The current study collected data on demographic factors (gender and age), SES (educational attainment and financial difficulties), living alone, marital status, health behaviors (smoking and drinking), health status (CMCs and pain intensity), and depressive symptoms. 

#### 2.5.1. Dependent Variable

Depressive Symptoms. This study used the 15 item-Short Geriatric Depression Scale (GDS) to evaluate depression [54,68]. Responses were on a “yes” or “no” scale. A summary score was calculated with a potential range between 0 to 15, in which a higher score indicated more depressive symptoms. The GDS-Short form has excellent reliability and validity, and it has been extensively used to measure depression among older adults in both clinical and community settings [54,68].

#### 2.5.2. Independent Variable

Demographic Factors. Gender was a dichotomous variable (1 female, 0 male), while age was treated as an interval variable.

Socioeconomic Status (SES). Educational attainment and financial difficulties were covariates in this study. Educational attainment was operationalized as an interval level variable (years of schooling), with higher scores indicating higher educational attainment. Financial difficulties were measured using three items (α = 0.923) that asked how often the participant did not have enough money for essential needs like food, rent/mortgage, clothes, and utility bills. Responses of each item were on a five-point Likert-scale (1 never to 5 always). We built a sum score with a range between 3 and 15, with a higher score reflecting more financial difficulties (lower SES).

Living arrangement and marital status. Living arrangements was a dichotomous variable (1 living alone, 0 living with someone else). Marital status was a dichotomous variable (1 married, 0 not married).

Health Behaviors (Smoking and Drinking). Participants were asked about their current smoking and drinking status, such as “How would you describe your cigarette smoking habits?” and “Do you drink alcohol?” Possible responses to the first question included never smoked, previously smoked, and current smoker. Possible responses to the second question were yes or no. These two variables were operationalized as dichotomous variables (1 drinker, 0 non-drinker; 1 ever smoker, 0 never smoker).

Chronic Medical Conditions (CMCs). Participants were asked if a physician had ever told them that they have any of the following 11 CMCs: hypertension, heart disease, diabetes, lipid disorder/hypercholesterolemia, cancer, asthma, osteoarthritis, thyroid disorder, chronic obstructive pulmonary disease, rheumatoid arthritis, and gastrointestinal disease. While self-reports provide valid information regarding CMCs, some measurement bias is also expected [69]. 

Pain Intensity. We measured pain intensity by four subscales of the McGill Pain Questionnaire-Short Form 2 (MPQ-SF-2) [70,71,72,73]. Participants responded to 22 items that asked about their experience over the past week of various types of pain. Each item was on a 11-point numeric rating scale of the extent of each type of pain (0 none to 10 worst possible). The subscales of the MPQ-SF-2 include: (a) continuity (throbbing, cramping, gnawing, aching, heavy, and tender pain), (b) intermittence (shooting, stabbing, sharp, splitting, electric-shock, and piercing pain), (c) neuropathic nature (hot-burning, cold-freezing, itching, tingling or “pins and needles”, light touch, and numbness pain), and (d) affective domain (tiring/exhausting, sickening, fearful, and punishing/cruel pain). A total pain score was calculated based on averaging responses to all questions [70,71,72,73]. A higher score indicated more intense pain.

### 2.6. Statistical Analysis

SPSS 22.0 (SPSS Inc., Chicago, IL, USA) was used to conduct the data analysis. Frequency (%) and mean (SD) were reported to describe the sample. Pearson’s correlation was used to calculate the bivariate correlations in the overall sample. We applied linear regression models with depressive symptoms as the outcome, and independent variables were gender, age, educational attainment, financial difficulties, living alone, marital status, smoking, drinking, CMCs, and pain. These predictors were determined based on the literature review. Health insurance was not included as a variable in our regression model considering only six participants reported not having insurance. We reported b (correlation coefficients), standard error (SE), 95% confidence intervals (95% CI), and *p* values.

## 3. Results

### 3.1. Descriptive Statistics

A total of 740 economically disadvantaged AA adults 55 years or older entered this study. From this number, 266 were AA men and 474 were AA women. Table 1 describes the sample overall.

### 3.2. Bivariate Correlations

Table 2 shows the correlation matrix between all the study variables in the overall sample. As this table shows, age, financial difficulties, living arrangement, ever smoking status, drinking, pain intensity, and number of CMCs were all correlated with depressive symptoms.

As Table 3 shows, age, financial difficulties, smoking, CMCs, and pain intensity were associated with depressive symptoms. Gender, educational attainment, living arrangement, marital status, and drinking were not associated with depressive symptoms.

## 4. Discussion

The current study revealed that among this sample of AA older adults residing in an economically disadvantaged setting, age, financial difficulties, smoking, CMCs, and pain intensity were associated with depressive symptoms. However, gender, educational attainment, living alone, marital status, and drinking were not predictive of depressive symptoms. 

While the mean score on the 15-item Short GDS was 2.47, falling in the 0 to 4 range that does not typically indicate clinically significant depression [68], the standard deviation was relatively high at 2.77. This indicated considerable variation with some participants scoring very low and others high in depressive symptoms. The mean score therefore did not necessarily rule out the mental health needs of our sample. We should emphasize that in the current study we were interested in modeling the variability of depressive symptoms, rather than the clinical diagnosis of depression. Therefore, while our outcome may not have suggested depression itself, it did reflect the presence of depressive symptoms. While there are lower prevalence rates of depression in AAs than Whites [12,13], AAs report more depressive symptoms than Whites, even when the individual does not meet criteria for a clinical diagnosis [14,15,16,17]. Considering all of this, we found that social, behavioral, and health factors explain the variation of depressive symptoms within our sample. 

### 4.1. Gender, Age, and Depressive Symptoms

Contrary to what may be expected based on national prevalence rates [13], gender was not a significant predictor of depressive symptoms in this sample. This finding aligns with previous research which found no difference in the prevalence of depression between AA men and AA women [14]. Furthermore, a recent review of the literature on depressive symptoms in AA populations suggests that AA men and AA women are exposed to different socialization and stressors which could lead to different presentations of symptoms and disparities in the recognition and treatment of those symptoms in mental health settings [26]. Evidence has shown how there are linkages between depression and risks of developing specific CMCs, such as metabolic syndrome, among AA women [74]. The current findings lend credence to the idea that while depression has typically been focused on in women, the reality of gender differences in the experience of depressive symptoms may be less pronounced, especially in older AA populations. 

Age, as predicted, was associated with risk for depressive symptoms in the current sample. Despite some recent research that suggests a boosting effect of age in mental HRQOL for AA men and women [27], our findings were similar to other research that shows age is an important risk factor for depressive outcomes [3]. Due to the aging process, these individuals are likely to have neurological and physiological changes such as disruptions in endocrine, inflammatory, immune or cardiovascular functioning, onset of dementia, and increased functional impairments, all of which are risk factors for depression [75]. Furthermore, aging comes with social changes such as retirement, which are also associated with risk for depression [76]. Further research has demonstrated that older AA adults internalized stigma and negative attitudes toward mental health care can keep them from seeking treatment, presumably resulting in more negative outcomes [28]. Given how these sociodemographic factors may influence depressive symptoms differently in AA populations, these findings demonstrate age as a relevant factor for determining risk for depressive symptoms among this economically challenged AA sample.

### 4.2. SES and Depressive Symptoms

While research in broader populations has indicated that educational attainment is negatively associated with depressive symptoms [4], our findings demonstrate no significant relationship between educational attainment and depressive symptoms. This, however, aligns with recent research demonstrating that educational attainment does not provide the same protective benefits for AA populations as it does for Whites [32,33]. Moreover, AA older adults have lower levels of educational attainment, have higher rates of unemployment, and are more likely to live in segregated poverty-stricken neighborhoods [77]. Our findings therefore corresponded with the Diminished Returns theory, in which gains in SES indicators result in smaller health gains for AA populations [78].

Having financial difficulties, however, was an SES indicator that significantly predicted more depressive symptoms among older AA adults. As measured in this study, financial difficulties were indicated by how frequently participants did not have enough money for necessities such as food or rent. Given the disparity of AA adults who live below the poverty line [35], it can be expected that financial difficulties may have been an especially important predictor of depressive symptoms in our sample of older AA adults living in an economically disadvantaged area. Low SES can be considered a chronic stressor, which has been linked to poor social and environmental conditions [79]. AA older adults experience higher rates of life-course financial strain, which is significantly associated with depression, cognition, and disability, leading to poor health outcomes [80]. Beyond the greater prevalence of financial difficulties among AA populations, recent research has also demonstrated that financial difficulties are more strongly associated with major depressive episodes for AAs than for Whites [9]. The current study’s findings on these two SES indicators, therefore correspond with recent research demonstrating the diminished protective returns of educational attainment and the importance of financial difficulties when predicting depressive symptoms in AA adults.

### 4.3. Living Arrangements and Depressive Symptoms

The current study results indicated that neither living alone nor being married were significant predictors of depressive symptoms in a sample of older AA adults in an economically disadvantaged area. Marriage has been associated with lower depressive symptoms both broadly and within AA samples [6,41]. While living alone has been positively associated with depressive symptoms in older adults in prior research [5,37,38], less research has been conducted on how this living arrangement predicts depressive symptoms in AA populations specifically. In addition, older AA adults believe that they are not susceptible to mental health issues, such as depressive symptoms, even after they have been diagnosed [81]. This can result in a growing gap between services needed by this subset population and those that are provided. Though some research has indicated no predictive relationship between depressive symptoms and social affiliations for AA adults [44], other research has focused on the benefits of support specifically from religious institutions and family outside of the home [45]. It is possible that the social isolation and lack of belonging that typically affects older adults living alone or being unmarried [39,40] may be buffered by increased involvement with religious institutions and family among our AA older adult sample. 

### 4.4. Health Behaviors and Depressive Symptoms

Interestingly, in our sample, smoking was predictive of depressive symptoms while drinking was not. The relationship between smoking and depression has been widely demonstrated [46,47], as has the increased risk for depression associated with alcohol use or AUD [7,48]. Our findings which demonstrate no association between drinking and depressive symptoms in this older AA adult sample could be due to several factors. The current study did not measure alcohol dependency or AUD but only current drinking status. Those who said they currently drink may drink at a level that is not significant enough to reach a threshold for increased risk of depressive symptoms. Furthermore, as mentioned previously, White adults have a higher prevalence of drinking rates than AA adults [53], so drinking may be a more relevant determinant of depression in White samples. While other research has demonstrated that despite lower rates of drinking, AA adults are at higher risk for some alcohol-related issues [10], the current findings can only speak to the risk of depressive symptoms for AA older adults who classified themselves as current drinkers, regardless of the level of drinking. 

On the other hand, being a smoker, either currently or in the past, was related to increased risk of depressive symptoms, as was predicted based on a review of the literature [46,47]. While prior research demonstrates the relationship between smoking and depression [46,47], it stands to reason that smoking also places our sample at increased risk for lung cancer, which has the highest incidence rate among AA populations [50], as well as other CMCs and pain, which the current findings show were also predictive of depressive symptoms in our sample. Overall, increased awareness of high prevalence rates for both alcohol and cigarette smoking in this population is needed in order to direct efforts toward behavioral intervention. Various types of care facilities and health care providers should promote alcohol and cigarette cessation in this sample to lower morbidity risk, such as depressive symptoms. Future research should focus on the mediational relationship of these factors specifically within AA populations who are at risk for both mental and physical health disparities.

### 4.5. CMCs/Pain and Depressive Symptoms

The current findings indicating CMCs and pain intensity are determinants of depressive symptoms within this older AA adult sample adds to the literature attempting to clarify the often observed Black-White paradox, in which AA adults are exposed to more distress and CMCs but exhibit lower rates of depression or other mental health conditions [12,57]. Older adults, specifically AAs, face many challenges, including the management of both chronic medical illnesses and geriatric syndromes. While the Black-White paradox relates specifically to the diagnosis of depression, when depressive symptoms were taken into account, our results aligned with a variety of other research that has demonstrated higher rates of comorbid CMCs and depressive symptoms among AA adults [8,11,56,59]. The results further aligned with past research showing an association between pain intensity and increased depressive symptoms for AA adults compared to Whites [61]. 

Demonstrating the relationship between CMCs, pain intensity, and depressive symptoms in a sample of older AA adults in an economically disadvantaged setting lends more evidence for the need to focus on these physical health conditions as areas for intervention to reduce the risk of depressive symptoms as well. Symptoms of chronic illnesses can mask psychological symptoms, resulting in a delay of detection, care, and treatment of depression [82]. These individuals may initially present to health care providers with symptoms, such as lethargy, decreased appetite, or weight changes, all of which are common with certain CMCs such as cancer, diabetes, or thyroid disorder [83,84,85], but are also common symptoms of depression [86]. However, a meta-analysis of the literature revealed that when patients see non-psychiatric physicians concerning depressive symptoms, less than half are recognized for depression [87]. Furthermore, the odds of recognition are significantly lower for older patients [87]. Given the heightened rates of CMCs and the disparity in pain treatment and depression treatment in AA populations [8,11,18,62], understanding the relationship these conditions have with mental health is crucial. 

### 4.6. Limitations

This study is not without limitations that come inherently with the study design. Due to the cross-sectional nature, causal associations could not be inferred. We did not have data on history of depression, area level factors, and household income. Due to a lack of clinical records to validate CMCs or mental health diagnosis, a self-report measurement bias may have existed. The measures used in our study such as the 15-item short GDS or MPQ-SF-2 have been validated for use in similar samples [54,68,70,71,72,73]. Furthermore, evidence has supported the validity of self-report measures for drinking, smoking, and CMCs [69,88,89]. Despite this, measurement bias may be found in this study. The importance of the validity of measures for use in AA sample cannot be overstated as measures developed in majority White samples may not carry the same translational, conceptual, or metric equivalence in non-White samples [90]. In order to limit measurement bias, future studies should utilize medical chart review or administrative data, as well as other measures validated for use in older AA samples in order to replicate these findings. Finally, the non-random sampling reduced generalizability of the results. Despite these limitations, this study’s findings further the literature on determinants of depressive symptoms among AA older adults in economically disadvantaged urban settings. 

## 5. Conclusions

In summation, age, financial difficulties, smoking, CMCs, and pain intensity were associated with depressive symptoms in economically disadvantaged AA older adults in South Los Angeles. Future research should focus on uncovering more about how these factors relate to depressive symptoms in older AA adults, as well as other racial/ethnic and age groups. Given the limited resources in this economically disadvantaged area of Los Angeles, these factors can inform screening efforts to identify those at risk for depression as well as clinical interventions designed to reduce the risk of depressive symptoms for this population. 

## Figures and Tables

**Table 1:** Descriptive statistics in the pooled sample (*n* = 740).

	Mean	SD
Age	71.73	8.37
Educational Attainment	12.74	2.24
Financial Difficulties	8.92	5.46
Number of CMCs (Multimorbidity)	3.86	1.86
Depressive symptoms	2.47	2.77
	*N*	%
Male		
No	474	64.1
Yes	266	35.9
Live Alone		
No	294	39.7
Yes	446	60.3
Ever Smoker		
No	347	47.0
Yes	391	53.0
Drinking Alcohol		
No	481	65.1
Yes, Rarely	79	10.7
Yes, Occasionally	132	17.9
Yes, Daily	47	6.4
Married		
No	640	86.5
Yes	100	13.5
Living Alone		
No	294	39.7
Yes	446	60.3

SD; standard deviation.

**Table 2:** Bivariate correlation matrix in the pooled sample and by gender.

	1	2	3	4	5	6	7	8	9	10	11
All											
1 Age	1.00	−0.08 *	−0.18 **	−0.31 **	0.00	0.06	−0.27 **	−0.21 **	−0.23 **	−0.02	−0.25 **
2 Gender (Male)		1.00	−0.11 **	0.07	0.12 **	−0.09 *	0.17 **	0.04	−0.07	−0.12 **	0.02
3 Education			1.00	−0.09 *	0.06	−0.04	−0.06	0.09 *	−0.01	−0.09 *	−0.07
4 Financial Difficulties				1.00	−0.07 *	0.12 **	0.31 **	0.16 **	0.36 **	0.22 **	0.43 **
5 Married					1.00	−0.41 **	−0.06	−0.08 *	−0.08 *	−0.02	−0.07
6 Living Alone						1.00	0.12 **	0.08 *	0.16 **	0.09 *	0.12 **
7 Smoking							1.00	0.24 **	0.14 **	0.13 **	0.23 **
8 Drinking								1.00	0.12 **	0.01	0.08 *
9 Pain									1.00	0.46 **	0.46 **
10 CMCs (Multimorbidity)										1.00	0.32 **
11 Depressive Symptoms											1.00

* *p* < 0.05, ** *p* < 0.01. 3.3. Linear Regression.

**Table 3:** Determinants of depressive symptoms in economically disadvantaged AA older adults.

	b	SE	B	95% CI		*p*
Age	−0.03	0.01	−0.10	−0.06	−0.01	0.003
Gender (Male)	0.07	0.19	0.01	−0.30	0.44	0.709
Educational Attainment	−0.05	0.04	−0.04	−0.13	0.03	0.251
Financial Difficulty	0.12	0.02	0.25	0.09	0.16	0.000
Married	−0.06	0.28	−0.01	−0.61	0.48	0.822
Living Alone	0.17	0.20	0.03	−0.21	0.56	0.384
Ever Smoker	0.38	0.19	0.07	0.00	0.75	0.050
Drinking Alcohol	−0.08	0.08	−0.03	−0.25	0.08	0.307
Pain	0.34	0.05	0.28	0.25	0.43	0.000
CMC (*n*)	0.18	0.05	0.12	0.08	0.29	0.001
Constant	2.83	1.16		0.55	5.12	0.015
